# Do Subjects from Different Occupational Groups Experience Dental Fear and Anxiety Equally?

**DOI:** 10.3390/medicina60040674

**Published:** 2024-04-21

**Authors:** Eglė Slabšinskienė, Karolina Radlinskaitė, Aistė Kavaliauskienė, Ingrida Vasiliauskienė, Jūratė Zūbienė, Kristina Saldūnaitė-Mikučionienė, Apolinaras Zaborskis

**Affiliations:** 1 Department of Oral Health and Paediatric Dentistry, Faculty of Odontology, Medical Academy, Lithuanian University of Health Sciences, A. Mickevičiaus 9, LT-44307 Kaunas, Lithuania; radlinskaitek@gmail.com (K.R.); ingrida.vasiliauskiene@lsmu.lt (I.V.); jurate.zubiene@lsmu.lt (J.Z.); kristina.saldunaite@lsmu.lt (K.S.-M.); 2 Department of Orthodontics, Faculty of Odontology, Medical Academy, Lithuanian University of Health Sciences, A. Mickevičiaus 9, LT-44307 Kaunas, Lithuania; aiste.kavaliauskiene@lsmu.lt; 3 Department of Preventive Medicine & Health Research Institute, Faculty of Public Health, Medical Academy, Lithuanian University of Health Sciences, A. Mickevičiaus 9, LT-44307 Kaunas, Lithuania; apolinaras.zaborskis@lsmu.lt

**Keywords:** dental fear, dental anxiety, prevalence, predictors, physicians, teachers, industry workers, artists, multi-group analysis

## Abstract

*Background and Objectives:* Research into the relationship between occupation and dental fear and anxiety (DFA) is scarce. This exploratory study aimed to compare the level of DFA and its association with its predictors amongst adults from different occupational groups. *Materials and Methods:* A cross-sectional study with 422 respondents from four occupational groups (physicians, teachers, industry workers, and artists) was carried out. A questionnaire on previous dental experience using the Dental Anxiety Scale (DAS), Dental Fear Survey (DFS), and Self-Esteem Scale was self-administered electronically. The data analysis involved descriptive statistics and structural equation modeling (SEM). *Results:* The DFA levels differed significantly across the occupational groups, with the lowest mean scores among physicians (DAS = 9.29 (SE 0.39); DFS-1 = 14.67 (0.63); DFS-2 = 33.94 (1.69)) and the highest mean scores among artists (DAS = 10.74 (0.38); DFS-1 = 17.19 (0.71); DFS-2 = 41.34 (1.92)). A significant impact of self-esteem on DFA was observed among physicians, teachers, and artists, but not among industry workers. Multi-group analysis with SEM revealed differences in the variable association (Chi-squared = 53.75; df = 21; *p* < 0.001), thus rejecting the hypothesis of the same mechanism underlying DFA across occupational groups. *Conclusions:* Individuals from various occupations experience DFA at different levels, and there are different mechanisms underlying their DFA. These findings can provide valuable insights for dental practitioners in developing tailored approaches to reduce the feeling of DFA of their patients.

## 1. Introduction

Despite advances in dental science and dental treatment technology, even adult patients still experience dental fear or dental anxiety. By definition, “dental fear” is a biological response to known threatening stimuli associated with dental treatment, whereas “dental anxiety” is a vague, unpleasant emotional state experienced by dental patients [1]. Regardless of origin, both of these terms are conceptualized as a strong negative patient reaction related to dental treatment and are, therefore, often used synonymously or even combined into the single term “dental fear and anxiety” (DFA) [1,2,3,4]. The literature suggests that DFA can originate in childhood, adolescence, or even later in life and is, therefore, common across the lifespan [5]. There is evidence that DFA, as a “vicious cycle”, has negative impacts on dental care and clinical and subjective oral health [2,6,7,8]. In meta-analyses, the global pooled prevalence of DFA has been estimated and found to be high in both children (23.9%; 95% CI: 20.4, 27.3) [9] and adults (13.8%; 95% CI: 9, 21; with 11.2% with high and 2.6% with severe DFA) [2]. In Lithuania, it was found that 56.1% of people were afraid of dental treatment, and 9.5% of them experienced a very high degree of fear of dental treatment [10]. 

There are many factors that may have an impact on the presence of DFA [11]. Its prevalence varies widely due to individual characteristics, such as age, gender, education, previous dental experience [1,2,3,4,12], and cultural, social, and economic differences between populations [13,14,15], as well as study design and the instruments used to measure DFA [2,4,16,17]. 

Numerous studies have detected that younger people are generally more fearful of dental treatment compared to older people [2,18,19,20]. Females were also more prone to report having DFA while anticipating treatment [2]. There are studies suggesting that DFA is more common in individuals who are single than in individuals in marriage [21,22,23]. Previous research has also shown that education plays an important role in the manifestation of DFA. It is usually seen that individuals with higher education and health literacy levels experience less DFA compared to their corresponding counterparts [24,25,26]; however, a few other studies have shown slightly different results [15,27,28]. Likewise, many papers have emphasized a positive relation between different socioeconomic status and dental fear. A higher socioeconomic status can be linked to less dental fear or dental anxiety, as opposed to individuals who have poor socioeconomic status [15,24,29,30]. This trend can be attributed to level of education since individuals with more years of education are usually at higher socioeconomic levels [31].

A number of studies have focused on the association between DFA and self-esteem [13,32,33,34,35,36,37]. Enhanced self-esteem is believed to act as a buffer against the development of anxiety [37]; trait anxiety, in particular, is consistently related to low self-esteem [38,39]. The effect of self-esteem on consequent anxiety appears to be stronger than the effect of anxiety on self-esteem [40], indicating that the two are strongly related.

Finally, according to the conceptual model of Stein Duker et al. [1], the literature shows evidence for relationships between DFA and poor oral health [26], irregular attendance at a dentist’s office [26,41], dental behavior management problems, a poor experience at a dental office [42], and the need for treatment using pharmacological methods [43].

People with the same education and socioeconomic level are usually brought together in society by the same profession or occupation. It was also established that representatives of the same profession have similar psychological values and personality traits. The most typical personality trait for physicians was awareness [44], for teachers—extraversion [45], and for artists—openness to experience [46]. A study conducted in Germany [47] found a positive correlation between the prevailing personality dimensions and the regularity of visits to the dentist; people whose personality was dominant extraversion, conscientiousness, or openness to experience were more likely to visit the dentist regularly than those in which sincerity or neuroticism predominated.

To date, very few studies have been conducted regarding the relationship between occupation and DFA. Some studies show a link between one’s occupation and DFA; however, they do not evoke the importance of certain personality traits that are possibly common in individuals of certain occupational groups. For example, a national cross-sectional study in France [48] showed that farmers and low-skilled workers were significantly more anxious than executives and shopkeepers, which is possibly related to the difference in education levels. Another study showed that individuals of certain professions have very particular personalities and character profiles and very different emotional temperaments [49]. Therefore, an individual’s temperament and way of reacting to various situations in life determines their level of DFA and behavior during dental visits [24]. However, even though many psychosocial variables can be associated with DFA, there are almost no studies that demonstrate a direct link between occupational status and the level of DFA. Therefore, this exploratory study aims to compare the level of DFA and its association with the predictors amongst adults from different occupational groups. Accordingly, we tested the hypothesis that the same mechanism underlies DFA across different occupational groups. Deeper insight into how occupation may influence DFA can help dental practitioners in developing tailored approaches to their patients’ care.

## 2. Materials and Methods

### 2.1. Study Design and Participants

This study adopted a cross-sectional design. Using G*Power 3.1 software (University of Dusseldorf, Dusseldorf, Germany) [50], based on a *t*-test, a minimum sample of *n* = 400 participants (100 participants in each occupational group) was calculated to detect differences between any two independent means, given α = 0.05, power = 0.8, and an effect size of 0.4 (less than medium).

An anonymous electronic survey was conducted to obtain responses from physicians, teachers, artists, and industry workers. To this end, an invitation to participate in the survey and an anonymous electronic questionnaire were placed on Facebook pages of the specific social network groups “Physicians of Lithuania/Lietuvos medikai”, “Teachers/Mokytojai”, “Amateur and professional actors/Aktoriai mėgėjai ir profesionalai”, “Artists of Lithuania/Lietuvos menininkai”, “Mondelez Lietuva”, and “Continental Lithuania”. All representatives of the selected occupational groups could participate in the study, regardless of their gender or age. With the consent of the network administrators, the survey was conducted from September 2021 to January 2022. During this period, data were obtained from 422 respondents, of which 162 were physicians, 106 were teachers, 62 were artists, and 92 were industry workers. Detailed characteristics of the respondents are presented in the Results (Section 3.1).

### 2.2. Measures

A self-administered electronic questionnaire was used to collect the data. The background data included the respondents’ gender, age, occupation/profession, marital status, education, and personal income. Only the questionnaires of the respondents of four occupations (physicians, teachers, industry workers, and artists) were registered. In the analyses, age was scored as ≤40 years and >40 years, marital status was scored as married or unmarried (all remaining conditions), education level was scored as higher (university) education and less than higher, and personal income was scored as ≤900 EUR/month and >900 EUR/month. Next, the respondents were asked to answer six questions (V1 to V6) related to their oral health and previous dental experience: (1) frequency of visits to the dentist (response options: 1—once a year or more often; 2—less often); (2) decayed teeth in need of treatment (1—yes; 2—no/does not know); (3) ever had a toothache (1—yes; 2—no); (4) satisfied with the state of their teeth (1—yes; 2—no); (5) had an unpleasant experience at the dentist’s office (1—yes; 2—no); (6) would like dental treatment procedures to be performed under sedation (1—yes; 2—no).

DFA was measured using two scales, the Dental Anxiety Scale (DAS) [51,52] and the Dental Fear Survey (DFS) [53,54]. Both scales have been adopted and tested for over 30 years in dental and psychological research worldwide [4,55], and they have been validated for the Lithuanian population [56].

The DAS scale allows for the assessment of the level of anxiety related to dental treatment [51,52]. The scale consisted of four questions, and the answers made it possible to assess the individual’s subjective reaction to certain situations related to dental treatment, such as waiting for an appointment, cleaning plaque, and drilling teeth. For each question, the following simple Likert scoring categories were assigned for whether the respondent was 1—“not anxious”; 2—“slightly anxious”; 3—“fairly anxious”; 4—“very anxious”; or 5—“extremely anxious”. Cronbach’s alpha of this scale was equal to 0.931. The items were summed to derive the total score, which ranged from 4 to 20, with the highest score denoting a high level of anxiety. The severity of dental anxiety could also be assessed according to the median value: a sum score <10 meant a low level of anxiety, and a sum score ≥10 meant a high level of anxiety.

The DFS scale was chosen for the assessment of the respondents’ dental fear [53,54]. Its original version was a 27-item questionnaire [53]. In the current study, we followed Raciene’s study [56], where the number of items was reduced to 24. Cronbach’s alpha for these items was equal to 0.973. The DFS assesses different dimensions of dental fear [57]. Based on the context of the questions and the form of the answers to the questions, in this study, we decided to analyze the two dimensions of the DFS separately. The first 8-item dimension (DFS-1) describes the subject’s avoidance of dental treatment due to fear and symptoms occurring during dental procedures. The answers were evaluated on a five-point Likert scale from 1 to 5, where 1—“never” or “not at all”; 2—“once or twice” or “a little bit”; 3—“several times” or “a little”; 4—“frequently” or “on average”; 5—“always” or “very”. The questions of the second 16-item dimension (DFS-2) describe the level of the subject’s perceived fear or other unpleasant feelings caused by visits to the dentist’s clinic and dental procedures. The answers were scored as follows: 1—“not at all”; 2—“a little bit”; 3—“a little”; 4—“moderately”; 5—“a lot”. The sum scores could range from 8 to 40 points and from 16 to 80 points for DFS-1 and DFS-2, respectively, with the highest score indicating a high level of dental fear. We used the sum score median values of the DFS-1 (14 points) and the DFS-2 (30 points) to divide the subjects into a fearful and a non-fearful group.

The questionnaire also comprised the 10-item Lithuanian-translated version of the Rosenberg Self-Esteem Scale [58]. Responses for these questions were recorded on a four-point Likert scale varying from 0 (“strongly agree”) to 4 (“strongly disagree”). Controlling for five negatively worded statements, the total self-esteem score was calculated. It varied from 0 to 30, with the highest value denoting the lowest level of self-esteem.

### 2.3. Statistical Analysis

First, we calculated the descriptive statistics of the variables of interest to characterize the study sample. The proportions and mean values of the analyzed variables were estimated for the data of each occupational group and for the total sample with weighting data by the sample size of each occupational group to ensure that the sample was representative of the general population. A comparison of the means of the DFA scores among the occupational groups of respondents was performed using features of multivariate general linear models: an ANOVA test; a calculation of the marginal means (estimations that are obtained by adjusting the data for other factors); and a post hoc comparison of the means among groups of respondents using, for instance, a Bonferroni test. This analysis made it possible to determine the strength of the relationship between the DFA measures and other variables and the “direction” (positive or negative) of the relationship. In these, as well as in the subsequent analyses, the significance level was set at *p* < 0.05. Descriptive analyses were performed with SPSS (version 21.0; SPSS Inc., Chicago, IL, USA, 2012).

Second, we developed a structural equation model (SEM) to assess the pathways between the overall (latent) DFA and the variables related to it. An example of the path diagram of the studied associations is presented in the Results (Section 3). Using path analysis methodology [59,60,61], the model examined the hypothesized causal relationships of DFA with oral health and dental experience, as well as with the self-esteem of the respondents, adjusting the data for age, marital status, and personal income (gender and education level were not included in the model because, in some occupational groups, these variables had only one value). In this model, the latent variable DFA was considered a dependent (endogenous) variable. It combined the scores of the three scales and the respondents’ answers to questions V1–V6. The sociodemographic variables (age, personal income, and marital status) were considered independent (exogenous) variables. Self-esteem was considered a direct predictor of DFA, as well as a mediating variable between the sociodemographic variables and DFA. In order to improve the overall model fit, covariance was added between variables V2 (decayed teeth that need to be treated) and V4 (satisfied with the condition of teeth). The use of modification indices helped to identify other covariances in several occupational groups. The unidirectional relationships provided standardized regression coefficients (*β*), and the bidirectional relationships provided correlation coefficients (*r*), showing the strength of the association between the connected variables. Squared multiple correlations (*R*^2^) were displayed for each endogenous variable, which is the proportion of variable variance that is accounted for by its predictors. The χ^2^ statistic related to the degree of freedom (χ^2^/df) was used to assess the magnitude of the discrepancy between the sample and the fitted covariance matrices, where *p* > 0.05 indicated that the model and the data were consistent. The model fit was also evaluated using the root mean square error of approximation (RSMEA) and other goodness-of-fit statistics: the comparative fit index (CFI); the Tucker–Lewis index (TLI); and the incremental fit index (IFI). Note that the RMSEA statistic measures how far our model is from a perfect model, while, on the contrary, the CFI, TLI, and IFI compare the fit of a hypothesized model with that of a baseline model (i.e., a model with the worst fit) [41]. An RSMEA value lower than 0.09 and CFI, TLI, and IFI values higher than 0.9 indicate good model fit to the real data [61,62]. SEM analysis was performed using AMOS 21 (SPSS Inc., Chicago, IL, 2012) [60].

Finally, the invariance of the DFA models in different occupational groups, an essential part of the present data analysis, was tested by applying SEM multi-group analysis. This analysis was important to examine whether the relationship between each predictor and DFA differed across the study groups. Basically, the invariance of regression weights, covariances, intercepts, and measurement errors across groups can be verified using multi-group analysis. In order to verify the invariance of the SEM model structure, such as equal regression weights across groups, the model was constrained for this structure (identical parameter values were set for all groups). The invariance of this model structure was then found to be satisfied when the difference in χ2 between the unconstrained and constrained models was insignificant [60].

## 3. Results

### 3.1. Descriptive Analysis of Occupational Groups

The DFA questionnaires were completed by 422 respondents, including 162 (38.4%) physicians, 106 (25.1%) teachers, 92 (21.8%) industry workers, and 62 (14.7%) artists. The respondents’ ages ranged from 18 to 77 years, with a mean of 38.8 years (SD = 12.9). There was a predominance of females (82.7%) over males (17.3%) in the total sample. In total, 80.3% of the respondents showed a higher education level versus 19.7% of the respondents who, in turn, showed a low education level. The proportions of married and unmarried respondents and the proportions of respondents by the selected monthly income criterion were similar.

Table 1 presents the sociodemographic characteristics of the respondents in the four selected occupational groups. Clear differences in the sociodemographic characteristics among the groups can be seen. The occupational groups of physicians and teachers were exclusively composed of women and persons with higher education. The average age of the teachers was 47.5 years old, which was significantly older than the persons of the other occupational groups. The group of artists stood out with the lowest percentage (25.5%) of married persons. The highest percentage of individuals with a higher monthly income was found among the physicians, while the lowest percentage of such individuals was observed among the artists (71.4% vs. 32.1%; *p* < 0.05).

When comparing the occupational groups, differences were also found in terms of the oral health and dental experience of their representatives (Table 2). It was observed that the physicians, compared to the representatives of other occupational groups, regularly visited the dentist to a greater extent (63.8%) and were satisfied with the condition of their teeth (69.5%); consequently, they were less likely to have decayed teeth that had to be treated (21.9%) or to have a toothache (83.0%). According to these characteristics, the artists were the opposite of the physicians. In addition, the highest percentage of persons who claimed to have had an unpleasant experience at the dentist’s office (79.2%) and would like dental treatment procedures to be performed under sedation (41.5%) was determined in the occupational group of artists.

**Figure 1:**
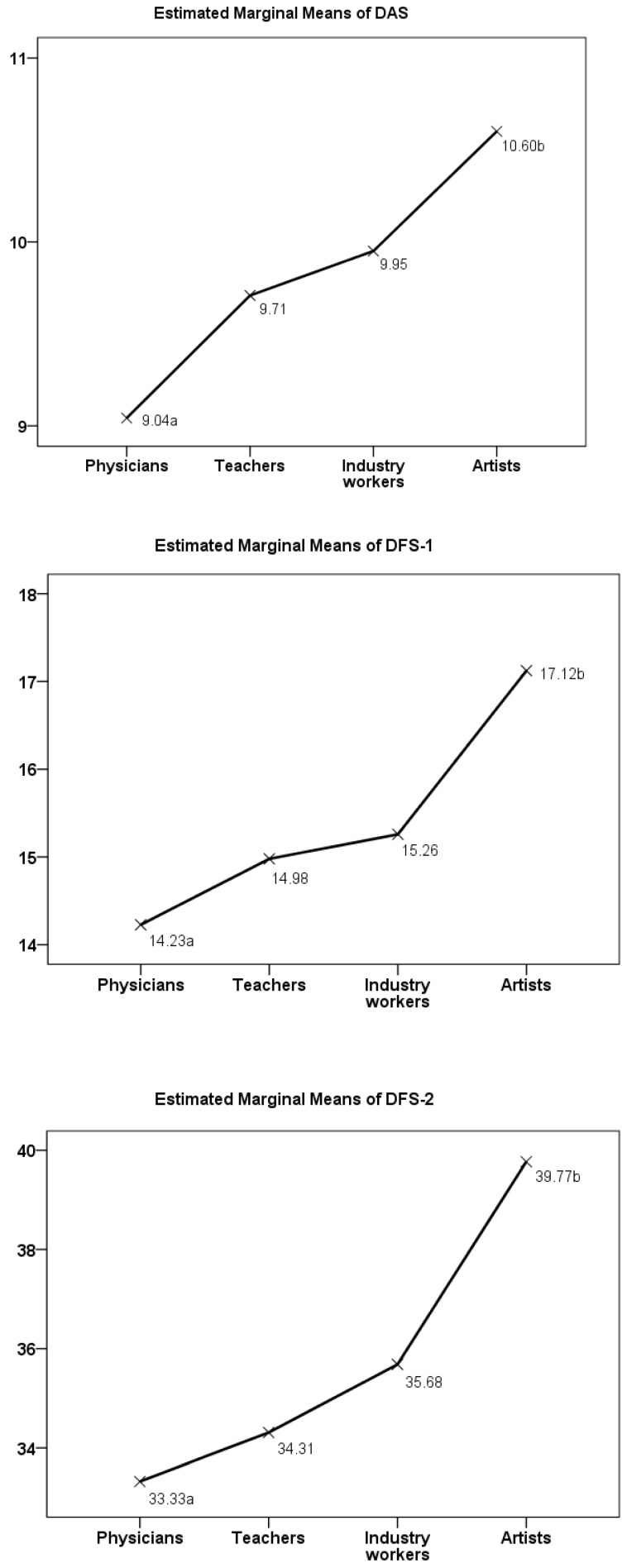
Marginal means of DFA measures estimated using multivariate general linear analysis. Data were adjusted for age, marital status, and personal income at Rosenberg’s self-esteem score of 19.58. Different subscripts of mean values (a, b) denote occupational groups whose estimations differ significantly from each other at the 0.05 level (Bonferroni test).

The data in Table 3 allow for a comparison of the crude descriptive statistics of the DFA measures among the occupational groups. All DFA measures, when comparing their averages or percentages of dichotomized variables, differed significantly (F- and Chi-squared tests) among the occupational groups. Significant differences in the means were seen between the physicians and artists, and also between the teachers and artists, except for the DAS. Differences in the mean statistics of the DFA measures between the physicians and artists were also revealed in the multivariate general linear analysis, adjusting data for age, marital status, personal income, and Rosenberg’s self-esteem score (Figure 1).

### 3.2. Associations among Variables

For the entire sample, the crude values of Pearson’s correlation coefficients between the DAS and DFS-1, the DAS and DFS-2, and the DFS-1 and DFS-2 were 0.814, 0.830, and 0.784, respectively (all correlations significant at *p* < 0.001).

**Figure 2:**
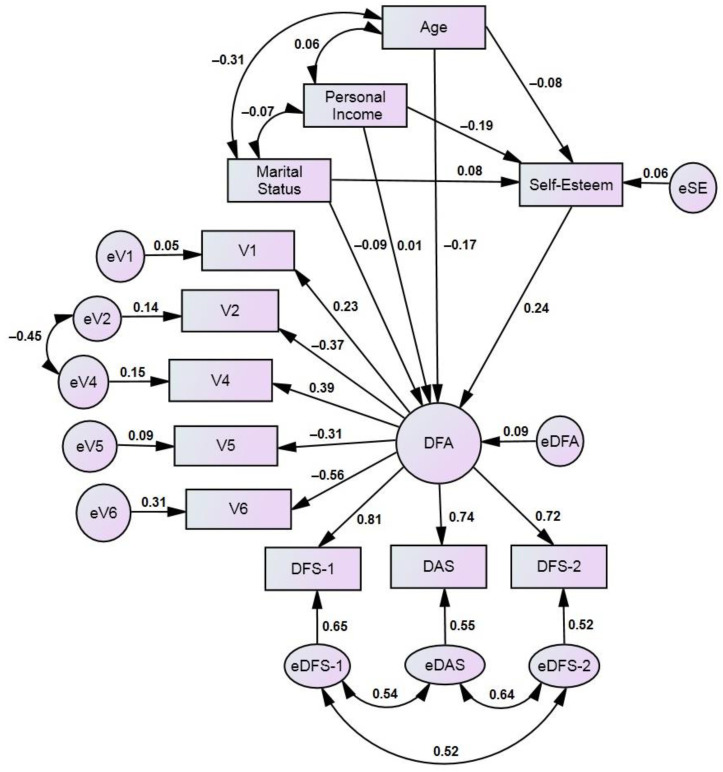
Path diagram with standardized estimates of the model, entire sample (*n* = 422). DFA: latent variable of dental fear and anxiety; DAS: Dental Anxiety Scale; DFS: Dental Fear Survey; V1: frequency of dental visits; V2: self-reported decayed teeth; V4: satisfied with the teeth; V5: unpleasant dental experiences; V6: requests for dental sedation.

A detailed analysis of the associations between the DFA measures and the variables that affect DFA was conducted using the SEM approach. A visualization of the model is presented in Figure 2.

A latent DFA variable integrated three DFA measures (DAS, DFS-1, and DFS-2). In addition, the latent variable was associated with five subjects’ oral health and dental experience variables (V1 to V6, but variable V3 (“Ever had a toothache”) was not used since most of the subjects had experienced this disorder). It was considered that age, personal income, and marital status affected DFA both directly and through self-esteem. Thus, the latter variable is both a predictor of DFA and a moderator between sociodemographic factors and DFA. A multi-group analysis was performed to test the validity of the hypothesis about the equity of associations existing across the different occupational groups of respondents. The model fit statistics of the unconstrained model for the entire sample were as follows: χ^2^/df = 2.209 (*p* < 0.001); CFI = 0.964; TLI = 0.946; IFI = 0.965; RMSEA = 0.054 (90% CI: 0.039; 0.068).

Table 4 presents selected estimates from the multi-group and the entire sample analysis. A graphical visualization of these data is presented in Figure 2 and in Figure S1 of the Supplementary Materials. It can be seen that all three FDA measurements (DAS, DFS-1, and DFS-2) equally strongly and significantly determined the latent DFA value regardless of the respondent’s occupational group. However, several estimates of the SEM model differed noticeably among the occupational groups. First, in the entire sample, it was found that older people (>40 years) experienced lower DFA; however, the effect of age and other sociodemographic variables on DFA, as well as on self-esteem, was not the same among the occupational groups. Second, self-esteem had a significant effect on DFA (lower self-esteem correlated with a higher level of DFA) in the occupational groups of doctors, teachers, and artists but was negligible among industry workers. Third, infrequent visits to the dentist (variable V1), reporting decayed teeth in need of treatment (variable V2), and dissatisfaction with the state of their teeth (variable V4) significantly increased the DFA of physicians and teachers, while these disorders did not have a significant effect on the DFA of industry workers and artists. On the other hand, having an unpleasant experience that previously occurred in a dentist’s office (variable V6) significantly increased the DFA among industry workers and artists but not among physicians and teachers. However, in all occupational groups, the respondents who would prefer dental treatment procedures to be performed under sedation (variable V6) had higher DFA values. Finally, differences among the occupational groups were also observed in the correlations among the variables. An exceptional example was the correlation between personal income and age; among the respondents of industry workers, this correlation was significantly negative, while in the remaining occupational groups, this correlation was positive (older respondents reported higher incomes).

The presented differences in the associations among the variables, which emerged from multi-group analysis, allowed us to hypothesize that the nature and level of DFA in the different occupational groups are not the same. This hypothesis was tested by nested model comparisons assuming the unconstrained model to be correct. AMOS examined every pair of models in which one model of the pair can be obtained by constraining the parameters of the other. Table 5 presents several statistics for comparing a more constrained model with the unconstrained model. This AMOS output shows that the hypothesis about the same mechanism of the nature of DFA among the occupational groups was rejected under the smallest constraints (e.g., constant DFA loadings).

## 4. Discussion

The results of this study give a positive answer to the question raised in the paper’s title, concluding that subjects from different occupational groups experience dental fear and anxiety differently. Firstly, the conclusion was proven by comparing the averages and percentage expressions of the DFA measurements among the respondents of the different occupational groups. Significant differences in DFA were observed between the physicians, who had the lowest mean scores of DFA, and artists, who had the highest mean scores of DFA. Finally, multi-group analysis with SEM demonstrated differences in the associations among the variables, thus rejecting the hypothesis that the same mechanism underlies DFA across the occupational groups of the study participants.

There are numerous methods to assess dental fear and anxiety [4,63]. Looking for a multifaceted measurement of the problem, in this study, we chose two instruments (DAS and DFS), which allowed for assessing both dental fear and dental anxiety. Moreover, the DFS instrument was divided into two parts based on the different approaches to exploring DFA. All three measures confirm that individuals from various occupations experience DFA at different levels, with the lowest mean scores and prevalence of high DFA among the physicians, and the highest values of the corresponding assessments among the artists. This conclusion corresponds to the observation of psychologists that representatives of the same profession have similar psychological values and personality traits [44,45,46]. It was noticed that awareness and cognition are the most typical personality traits for physicians [44]. Individuals with such traits are less likely to have anxiety and phobias [64]. This is one of the explanations why, in our study, the lowest level of DFA was found among the physicians. In contrast, the scientific literature highlights that individuals with professions that require creativity, such as artists, can be associated with a more frequent occurrence of anxiety [65,66] and other mental disorders [67]. On the other hand, anxious people tend to be very intelligent, and they are usually creative, intuitive, emotional, empathetic, and amiable [66]. Thus, these data from the literature may explain why artists showed the highest level of DFA in our study.

Another finding of this study shows that individuals from various occupational groups differ not only at the level of DFA but also have different associations between DFA and its predictors. SEM multi-group analysis helped to investigate these associations by occupational group. There are scarce studies in the literature in this field; however, regarding age, marital status, and personal income, the study results can be compared to the findings published in the literature. For instance, generally, an inverse relationship between age and anxiety, i.e., anxiety decreases with advancing age due to improved coping skills, is described in the literature [19,20,21,27]. On the contrary, a study by Armfield et al. [68] found that the middle adulthood age group, i.e., those aged between 40 and 64 years, had almost twice the prevalence of a high degree of fear as the other age groups combined, suggesting that middle adulthood represents a period of change and is associated with physical decline and increased illness. In the present study, the significant inverse relationship between age and DFA was found only among the physicians and artists, including in the analysis of the data of the entire sample. Next, previous studies have reported that DFA is more common in individuals who are single than in individuals in marriage [19,20,21]. According to our data, it seems that married physicians and teachers, in contrast to the previous studies, were more likely to report higher scores of DFA. Considerable evidence has also been found showing that low socioeconomic status may be related to a higher risk of DFA [26,68,69]. This study indicates that personal income did not have a significant direct effect on DFA, but contrary to the literature, higher artist incomes were positively correlated with DFA scores. The results of our SEM modeling indicate that the real effect of income on subjects’ DFA must be assessed not only directly but also through the mediator of self-esteem, which, again, can exacerbate the scores of DFA.

Numerous studies show a positive association between self-esteem and oral health. By maintaining healthy teeth and gums, individuals can feel more confident and comfortable in social situations, improving their self-esteem and quality of life [70,71]. Since poorer oral health elicits higher levels of DFA [68,69], there could be some suggestions for a relationship between self-esteem and DFA, but this issue has not been studied previously in such an intensive manner. Exceptional studies among children [13,33,34] have found that dental anxiety is negatively related to self-esteem, and this relationship is significant among older children only. Similar results were obtained in the present study, which reports that DFA was negatively related to self-esteem, i.e., a higher level of DFA was associated with lower self-esteem. The association was highly significant in the occupational groups of doctors, teachers, and artists but negligible among industry workers. Such a difference across occupational groups may be explained by the fact that the highest percentage of respondents with less than a higher education was the industry workers, whose work is more likely to be less competitive and which does not require much concern for self-efficacy [72].

The results of the entire sample analysis demonstrate that DFA was significantly associated with oral health and previous dental experience; however, these associations were not uniformly expressed across the occupational groups. For instance, infrequent visits to the dentist, decayed teeth in need of treatment, and low satisfaction with the state of their teeth were significantly associated with the occurrence of DFA only among the physicians and teachers. One of the reasons for these associations could be that subjects with DFA are more likely to delay their dental appointments, deteriorating their oral health status, and are more likely to experience unsatisfaction due to their dental state [24]. The observed differences in the associations across the groups can be explained by the fact that the groups of physicians and teachers who participated in the study consisted mostly of women. It has been widely studied that women have a lower tolerance to pain and generally report higher levels of DFA [2,23]. In another example, it can be seen that a previous unpleasant experience at the dentist’s office was significantly related to DFA only among industry workers and artists, although physicians and teachers also had quite a few negative experiences. One of the possible explanations for this phenomenon may be that all/most patients in these groups have higher education. We did not have the opportunity to assess the relationship between DFA and education, but the literature [18,73,74] suggests that higher education leads to a reduction in dental anxiety because the patients with higher educational levels may have better oral health or visit the dentist more regularly. Another explanation for this phenomenon could be that those with higher levels of education and better economic circumstances often experience a greater sense of self-esteem, control, mastery, and ability to effectively overcome their life obstacles [31,32]. Our study found that individuals with dental anxiety, regardless of their profession, would likely prefer sedation during treatment. Similar results were obtained in a study conducted in London, during which it was observed that sedation was preferred mostly by patients who were afraid and rarely visited the dentist [75].

This study has a few limitations. First, the study was cross-sectional in nature and did not attempt to determine the causal order. We constructed a model that allowed for examining the hypothesized causal relationships of DFA with respondents’ sociodemographic characteristics, oral health, previous dental experience, and self-esteem. These relationships were assumed to be unidirectional, but this should be interpreted with caution. For instance, self-esteem was considered a direct predictor of DFA, as well as a mediator between sociodemographic variables and DFA. However, several studies have indicated that dental anxiety has pervasive psychosocial consequences, including, but not limited to, lower self-esteem [37,76]. Thus, it would be a mistake to conclude, based on these study results alone, that by increasing one’s level of self-esteem, individuals will experience, as an effect, lower DFA. The relationship of DFA with oral health, irregular attendance at a dentist’s office, a poor experience at a dentist’s office, and the need for sedative treatment (variables V1 to V6) are more examples of the problem of the directionality of the relationship. Although numerous previous studies [1,24] have pointed out these relationships, their nature and causal directions remain to be established [77].

The second limitation was some gender imbalance in the sample’s composition, which had a greater proportion of women. It seems that women are more likely to respond to surveys [78]. It was most unfortunate that this happened in the groups of physicians and teachers, thus limiting the extent of the conclusions. There was also an imbalance in the sample regarding education level. However, this limitation is justified because in Lithuania, all physicians and teachers must have a higher education.

Despite these limitations, we believe that the current findings provide further evidence regarding DFA in particular population groups. However, further empirical research is needed to elucidate the specific relationship between occupation and DFA. Some potential factors that could contribute to differences in DFA among occupations might include the following: work-related stress, flexibility of scheduling, exposure to dental settings, access to dental care, occupational hazards, etc. [79]. Occupational factors in the design of dental interventions and patient management strategies can lead to more personalized and effective approaches to reduce the feeling of fear and anxiety arising from dental treatment in patients.

## 5. Conclusions

Individuals from various occupations experience dental fear and anxiety (DFA) at different levels, with the lowest mean scores and prevalence of high DFA among physicians and the highest values of the corresponding assessments among artists. Multi-group analysis revealed differences in the associations among the variables, thus rejecting the hypothesis that the same mechanism underlies DFA across occupational groups. These findings can provide valuable insights for dental practitioners in developing tailored approaches to reduce the feeling of fear and anxiety arising from dental treatment in their patients.

## Figures and Tables

**Table 1:** Sociodemographic characteristics of respondents by their occupational group ^a^.

Characteristics	Physicians		Teachers		Industry Workers		Artists		Total		*p*-Value
	n	(%)	n	(%)	n	(%)	n	(%)	n	(%)	
Gender:											
Males	6	(5.7 *)	2	(1.9 *)	33	(31.4 ^+^)	32	(30.2 ^+^)	73	(17.3)	<0.001 ^c^
Females	99	(94.3 *)	104	(98.1 *)	72	(68.6 ^+^)	74	(69.8 ^+^)	349	(82.7)	
Age:											
Mean (SE)	37.4 *	(1.18)	47.5 ^+^	(1.05)	36.6 *	(1.20)	33.7 *	(1.17)	38.8	(0.63)	<0.001 ^b^
≤40 years	64	(61.0 *)	24	(22.6 ^+^)	70	(66.7 *)	79	(74.5 *)	237	(56.2)	<0.001 ^c^
>40 years	41	(39.0 *)	82	(77.4 ^+^)	35	(33.3 *)	27	(25.5 *)	185	(43.8)	
Education:											
Less than higher	0	(0 *)	3	(2.8 *)	51	(48.6 ^+^)	29	(27.4 °)	83	(19.7)	<0.001 ^c^
Higher	105	(100 *)	103	(97.2 *)	54	(51.4 ^+^)	77	(72.6 °)	339	(80.3)	
Marital status:											
Married	59	(56.2 *)	72	(67.9 *)	50	(47.6 ^+^)	27	(25.5 °)	208	(49.3)	<0.001 ^c^
Unmarried	46	(43.8 *)	34	(32.1 *)	55	(52.4 ^+^)	79	(74.5 °)	214	(50.7)	
Personal income:											
≤900 EUR/month	30	(28.6 *)	49	(46.2 ^+^)	51	(48.6 ^+^)	72	(67.9 °)	202	(47.9)	<0.001 ^c^
>900 EUR/month	75	(71.4 *)	57	(53.8 ^+^)	54	(51.4 ^+^)	34	(32.1 °)	220	(52.1)	

Notes. ^a^ Crude data with weighting by occupational groups; ^b^ F-test to check whether the means were equal among the occupational groups; ^c^ Chi-squared test to check whether the characteristics were equally distributed among the occupational groups. Different superscripts (*, ^+^ and °) denote occupational groups whose estimations differ significantly from each other at the 0.05 level (Bonferroni test).

**Table 2:** Oral health and previous dental experience of respondents by their occupational group ^a^.

Characteristics	Physicians		Teachers		Industry Workers		Artists		Total		*p*-Value ^b^
	n	(%)	n	(%)	n	(%)	n	(%)	n	(%)	
V1: Frequency of visits to the dentist:											
Once a year or more often	67	(63.8)	52	(49.1)	51	(48.6)	50	(47.2)	220	(52.1)	0.052
Less often	38	(36.2)	54	(50.9)	54	(51.4)	56	(52.8)	202	(47.9)	
V2: Decayed teeth in need of treatment:											
Yes	23	(21.9 *)	30	(28.3)	37	(35.2)	46	(43.4 ^+^)	136	(32.2)	0.006
No/does not know	82	(78.1 *)	76	(71.7)	68	(64.8)	60	(56.6 ^+^)	286	(67.8)	
V3: Ever had a toothache:											
Yes	88	(83.0 *)	101	(95.3 ^+^)	100	(95.2 ^+^)	104	(98.1 ^+^)	393	(92.9)	<0.001
No	18	(17.0 *)	5	(4.7 ^+^)	5	(4.8 ^+^)	2	(1.9 ^+^)	30	(7.1)	
V4: Satisfied with the state of the teeth:											
Yes	73	(69.5 *)	60	(56.6 *)	66	(62.9 *)	43	(40.6 ^+^)	242	(57.3)	<0.001
No	32	(30.5 *)	46	(43.4 *)	39	(37.1 *)	63	(59.4 ^+^)	180	(42.7)	
V5: Had an unpleasant experience at the dentist’s office:											
Yes	76	(72.4)	66	(62.3 *)	62	(59.0 *)	84	(79.2 ^+^)	288	(68.2)	0.006
No	29	(27.6)	40	(37.7 *)	43	(41.0 *)	22	(20.8 ^+^)	134	(31.8)	
V6: Would like dental treatment procedures to be performed under sedation:											
Yes	19	(18.1 *)	19	(17.9 *)	40	(38.1 ^+^)	44	(41.5 ^+^)	122	(28.9)	<0.001
No	86	(81.9 *)	87	(82.1 *)	65	(61.9 ^+^)	62	(58.5 ^+^)	300	(71.1)	

Notes. ^a^ Crude data with weighting by occupational groups; ^b^ Chi-squared test to check whether the characteristics were equally distributed among the occupational groups. Different superscripts (* and ^+^) denote occupational groups whose estimations differ significantly from each other at the 0.05 level (Bonferroni test).

**Table 3:** Measures of DFA by occupational group of respondents ^a^.

Measures	Physicians		Teachers		Industry Workers		Artists		Total		*p*-Value
Dental Anxiety Scale (DAS):											
Mean (SE)	9.29 *	(0.39)	9.51	(0.29)	10.13	(0.37)	10.74 ^+^	(0.38)	9.92	(0.18)	0.020 ^b^
<10 scores, *n* (%)	69	(65.7 *)	64	(60.4)	58	(55.2)	50	(47.2 ^+^)	241	(57.1)	0.045 ^c^
≥10 scores, *n* (%)	36	(34.3 *)	42	(39.6)	47	(44.8)	56	(52.8 ^+^)	181	(42.9)	
Dental Fear Survey (DFS-1):											
Mean (SE)	14.67 *	(0.63)	14.79 *	(0.54)	15.46	(0.61)	17.19 ^+^	(0.71)	15.53	(0.31)	0.016 ^b^
<14 scores, *n* (%)	62	(59.0 *)	55	(51.9)	55	(52.4)	43	(40.6 ^+^)	215	(50.9)	0.059 ^c^
≥14 scores, *n* (%)	43	(41.0 *)	51	(48.1)	50	(47.6)	63	(59.4 ^+^)	207	(49.1)	
Dental Fear Survey (DFS-2):											
Mean (SE)	33.94 *	(1.69)	32.89 *	(1.38)	36.58	(1.87)	41.34 ^+^	(1.92)	36.19	(0.88)	0.003 ^b^
<30 scores, *n* (%)	58	(55.2 *)	50	(47.2)	54	(51.4)	39	(36.8 ^+^)	201	(47.6)	0.045 ^c^
≥30 scores, *n* (%)	47	(44.8 *)	56	(52.8)	51	(48.6)	67	(63.2 ^+^)	221	(52.4)	

Notes. ^a^ Crude data with weighting by occupational groups; ^b^ F-test to check whether the means were equal among the occupational groups; ^c^ Chi-squared test to check whether the characteristics were equally distributed among the occupational groups. Different superscripts (* and ^+^) denote occupational groups whose estimations differ significantly from each other at the 0.05 level (Bonferroni test).

**Table 4:** Standardized regression coefficients (*β*) and Pearson’s correlation coefficients (*r*) of the SEM model in multi-group and entire sample analyses.

Estimates	Physicians		Teachers		Industry Workers		Artists		Entire Sample	
	*β*/*r*	*p*	*β*/*r*	*p*	*β*/*r*	*p*	*β*/*r*	*p*	*β*/*r*	*p*
Standardized regression coefficients:										
DAS ← DFA	0.61	<0.001	0.70	<0.001	0.79	<0.001	0.83	<0.001	0.74	<0.001
DFS−1 ← DFA	0.57	<0.001	0.84	<0.001	0.92	<0.001	0.92	<0.001	0.81	<0.001
DFS−2 ← DFA ^a^	0.59		0.73		0.57		0.72		0.72	
DFA ← Age	−0.47	<0.001	0.03	0.762	−0.03	0.777	−0.29	0.020	−0.17	0.005
DFA ← Marital status	−0.31	0.002	−0.22	0.032	−0.12	0.264	0.14	0.272	−0.09	0.136
DFA ← Personal income	0.12	0.189	0.12	0.250	−0.26	0.056	0.32	0.011	0.01	0.848
DFA ← Self−esteem	0.23	0.015	0.42	<0.001	0.03	0.780	0.40	0.002	0.24	<0.001
Self−esteem ← Age	−0.14	0.082	−0.04	0.664	−0.27	0.010	0.06	0.629	−0.08	0.118
Self−esteem ← Marital status	0.08	0.310	−0.04	0.713	0.11	0.244	0.34	0.011	0.08	0.119
Self−esteem ← Personal income	−0.21	0.007	−0.10	0.304	−0.49	<0.001	0.12	0.357	−0.19	<0.001
V1 ← DFA	0.43	<0.001	0.35	0.003	0.13	0.236	0.24	0.084	0.23	<0.001
V2 ← DFA	−0.45	<0.001	−0.61	<0.001	−0.21	0.089	−0.14	0.285	−0.37	<0.001
V4 ← DFA	0.67	<0.001	0.37	0.002	0.25	0.063	0.16	0.223	0.39	<0.001
V5 ← DFA	−0.18	0.062	−0.17	0.122	−0.51	0.009	−0.50	<0.001	−0.31	<0.001
V6 ← DFA	−0.42	<0.001	−0.43	<0.001	−0.39	0.018	−0.82	<0.001	−0.56	<0.001
Pearson’s correlation coefficients:										
Marital status ↔ Personal income	0.01	0.938	−0.13	0.185	0.14	0.176	−0.31	0.009	−0.07	0.165
Personal income ↔ Age	0.19	0.015	0.23	0.015	−0.41	<0.001	0.21	0.086	0.06	0.188
Marital status ↔ Age	−0.27	<0.001	−0.12	0.219	−0.26	0.012	−0.32	0.012	−0.31	<0.001
eDFS−1 ↔ eDFS−2	0.71	<0.001	0.60	0.033	0.85	<0.001	0.17	0.592	0.52	0.003
eDAS ↔ eDFS−1	0.73	<0.001	0.57	0.033	0.32	0.695	0.24	0.555	0.54	0.003
eDAS ↔ eDFS−2	0.77	<0.001	0.51	0.015	0.77	0.027	0.61	0.020	0.64	<0.001
eV2 ↔ eV4 ^b^	−0.22	0.049	−0.11	0.324	−0.70	<0.001	−0.71	<0.001	−0.45	<0.001
eV6 ↔ Marital status ^b^	−0.24	<0.001			−0.21	<0.001				
eV4 ↔ Age ^b^			0.31	0.002						
eV1 ↔ eV2 ^b^			0.41	<0.001						
eV4 ↔ Personal income ^b^							0.27	0.006		
eV5 ↔ Marital status ^b^							0.29	0.021		

Notes. DAS: Dental Anxiety Scale; DFS: Dental Fear Survey; other abbreviations are explained in Table 2 and Figure 2. ^a^ Variable with constrained regression weight; ^b^ additional correlations suggested by modification indices. See Figure 1 and Figure S1 for details.

**Table 5:** Nested model comparisons, assuming unconstrained model to be correct.

Model	Constraints	Difference in df	Difference in Chi-Squared	*p*-Value
Model 1	DFA loadings to DAS, DFS, and DBS; V1 to V6 are constant across occupational groups	21	53.75	<0.001
Model 2	All of the above, and intercepts in the equations for predicting measured variables of DFA, are constant across occupational groups	42	101.61	<0.001
Model 3	All the above, and the regression weights for predicting self-esteem and DFA by socio-demographic variables, are constant across occupational groups	63	160.82	<0.001
Model 4	All the above, and intercepts in the equations for predicting self-esteem and DFA by sociodemographic variables, are constant across occupational groups	69	177.38	<0.001

## Data Availability

The datasets are available upon request to the corresponding author.

## Supplementary Materials

The following supporting information can be downloaded at: https://www.mdpi.com/article/10.3390/medicina60040674/s1, Figure S1: Path diagrams with standardized estimates of the model by respondents’ occupational group: A: Physicians (*n* = 162); B: Teachers (*n* = 106); C: Industry Workers (*n* = 92); D: Artists (*n* = 62).
