# Supplementary material for: Do Subjects from Different Occupational Groups Experience Dental Fear and Anxiety Equally?
Source: Medicina (Kaunas). 2024 Apr 21;60(4):674. doi: 10.3390/medicina60040674 (PMC11051849; doi:10.3390/medicina60040674)
Supplement: Supplementary file 1 [file medicina-60-00674-s001.zip › medicina-2911725-supplementary.pdf]

A: Physicians

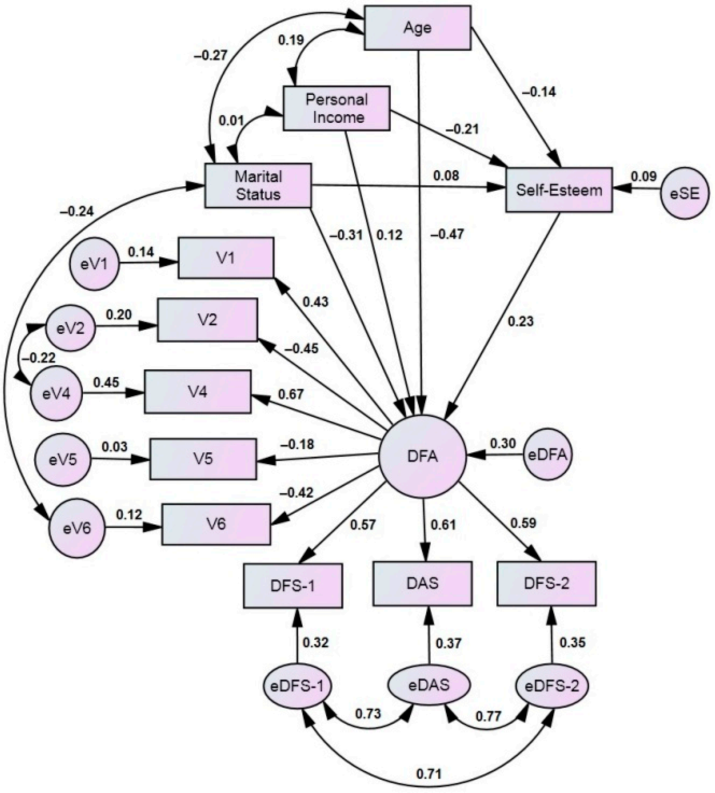

B: Teachers

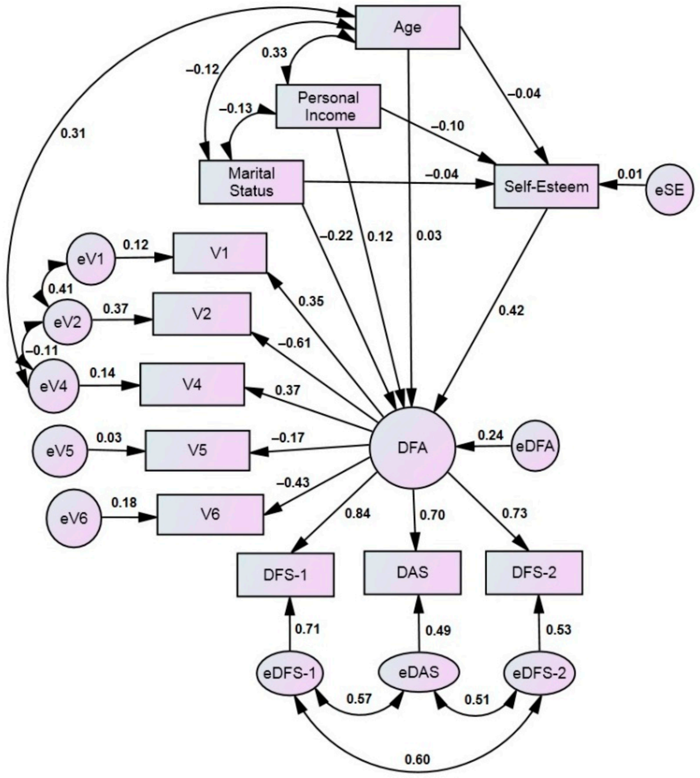

C: Industry workers

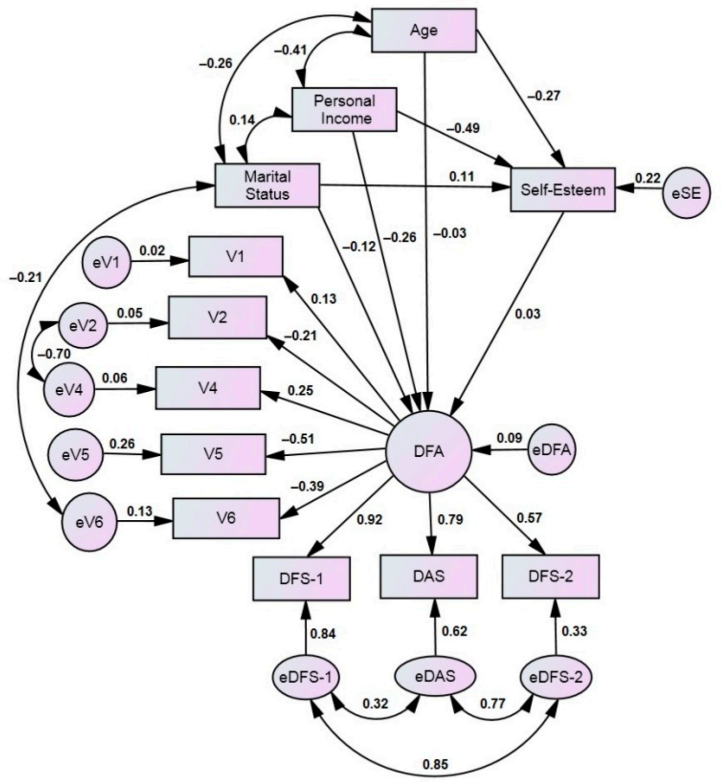

D: Artists

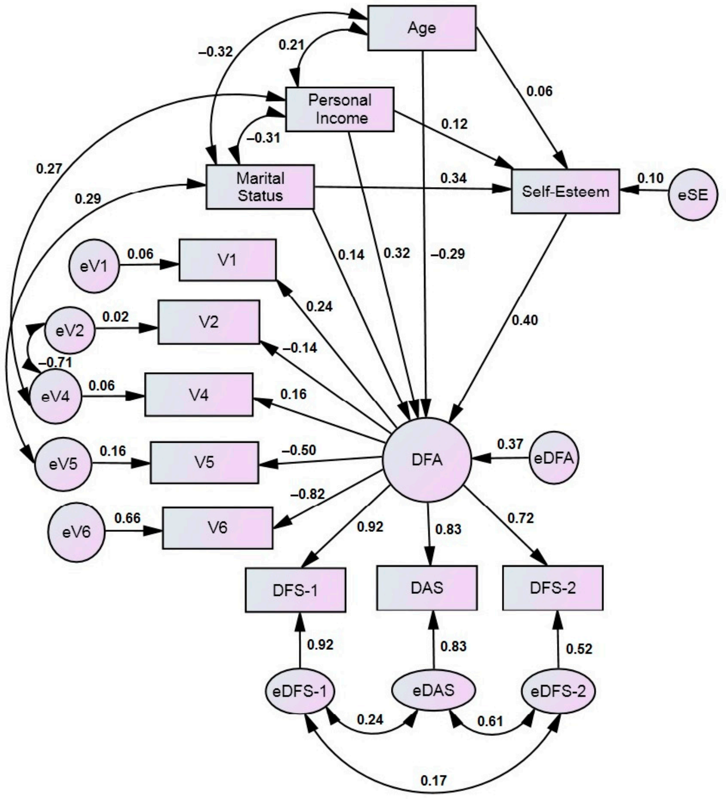

Figure S1. Path diagrams with standardized estimates of the model, by respondents' occupational group: A: Physicians (n=162); B: Teachers (n=106); C: Industry workers (n=92); D: Artists (n=62). DFA: Latent variable of Dental Fear and Anxiety; DAS: Dental Anxiety Scale; DFS: Dental Fear Survey;V1: Frequency of dental visits; V2: Self-reported decayed teeth; V4: Satisfied with the teeth; V5 unpleasant dental experiences; V6: requests for dental sedation.
